# Quality of life questionnaires in patients with acromegaly: a scoping review

**DOI:** 10.1186/s41687-026-01022-3

**Published:** 2026-03-11

**Authors:** Carlos E. Builes-Montaño, Jorge Eduardo Contreras-Saldarriaga, Oriana Arroyo-Ripoll, Jorge Hernando Donado-Gomez

**Affiliations:** 1 Internal Medicine Department, Endocrinology Section, Hospital Pablo Tobón Uribe, and Universidad de Antioquia, Medellin, Colombia; 2 Universidad de Antioquia, Medellin, Colombia; 3 Hospital Pablo Tobón Uribe, Universidad de Antioquia, Medellin, Colombia

**Keywords:** Quality of life, Patient reported outcome measures, Acromegaly, Growth hormone-secreting pituitary adenoma

## Abstract

**Introduction:**

Acromegaly is a rare disease caused by a pituitary adenoma in most cases. Physical changes, metabolic disturbances, osteoarticular symptoms, as well as psychological and social repercussions reduce the Quality of Life (QoL) of people suffering from the disease.

**Objective:**

To assess both generic and disease-specific instruments employed to capture the influence of acromegaly on patients’ QoL.

**Methods and analysis:**

This scoping review assessed the different tools used to evaluate QoL in people with acromegaly. This review followed the methods proposed by the Joanna Briggs Institute and is reported following the Preferred Reporting Items for Systematic Reviews manual and Meta-Analyses extension for Scoping Reviews.

**Results:**

Of a total of 198 studies, 30 questionnaires were identified and categorized by type (specific or generic) and number of domains assessed (physical health, psychological state, autonomy, and social relationships). Among these, 3 were specific-multidomain, 18 generic-multidomain, 3 specific-unidomain, 5 generic-unidomain, and only 1 had no domains. All were patient-reported outcome measures (PROM) used in various contexts. Optimal instruments were disease-specific, multidimensional, brief, easily administered, available in multiple languages, with psychometric validation in acromegaly patients and cross-cultural validation, and assessing treatment impact on QoL.

**Conclusions:**

This review explores the use of PROMs in assessing HRQoL in acromegaly patients, emphasizing the need for specific, validated, and culturally adapted tools addressing symptoms and treatment impact. We propose a novel domain-based classification to overcome existing limitations of instruments and improve their presentation in future studies. This study provides a synthesis that helps advance research and guide clinical practices around HRQoL in acromegaly management.

**Supplementary information:**

The online version contains supplementary material available at 10.1186/s41687-026-01022-3.

## Introduction

Acromegaly is a rare chronic disease caused mainly by a benign pituitary tumor that secretes excessive growth hormone (GH), leading to elevated insulin-like growth factor 1 (IGF-1) levels. Its global prevalence ranges from 18 to 137 per million people, with an annual incidence between 2 and 11 new cases per million inhabitants [1]. Clinically, acromegaly manifests as a dysmorphic syndrome with progressive bone growth and multiple comorbidities, including hypertension, glucose intolerance and diabetes, sleep apnea, respiratory disorders, heart failure [1, 2], and chronic rheumatologic conditions with joint deformities that cause pain and disability, often persisting even after biochemical control [3]. The disease also has psychological and social repercussions that substantially affect patients’ quality of life [4].

Traditional treatment goals focus on normalizing GH and IGF-1 levels, controlling tumor mass, and reducing comorbidities to lower mortality. However, due to the chronic nature and physical impact of acromegaly, patient-reported outcomes (PROs) are crucial to capture the broader consequences of the disease [5].

People with acromegaly usually report lower health-related quality-of-life (HRQoL) scores than those with other pituitary tumors [6–8] partly because of delayed diagnosis and treatment, which allow greater disease progression [9–12]. Even after achieving biochemical control, HRQoL often remains impaired due to persistent symptoms such as fatigue, headache, muscle pain, or cognitive dysfunction, and because some physical changes are irreversible [10, 13–15].

In acromegaly, health-related quality of life (HRQoL) has been assessed using both generic and disease-specific instruments [16, 17]. Generic tools may better identify health needs, allow comparison of HRQoL across diseases, and help estimate the economic impact of acromegaly [18–20]. while disease-specific instruments more accurately capture the condition’s unique impact and treatment effects [21]. HRQoL, defined as an individual’s subjective perception of health [22], has gained increasing importance in evaluating the effectiveness of care programs and interventions. Its assessment, together with other patient-centered measures, reflects the growing incorporation of patient-reported outcomes (PROs) into clinical research and healthcare decision-making [3, 23]. PROs provide valuable insights into patients’ experiences, improving communication with clinicians and aligning care with patient preferences and autonomy [24]. Despite advances in acromegaly treatment, QoL remains significantly impaired [3, 25], emphasizing the need to integrate HRQoL and PRO assessments into comprehensive disease management. Specific tools such as the AcroQoL [3, 26] have been developed for acromegaly, but their use may be limited by clinicians’ familiarity with generic instruments or the wider cultural validation of those tools. Given the rarity of acromegaly, a combination of generic and disease-specific instruments is frequently used in clinical and research settings [15]. Therefore, mapping the literature on these instruments is essential to understand how HRQoL is currently evaluated in people with acromegaly, identify existing gaps, and guide future research and clinical practice. This scoping review aims to systematically map and characterize the instruments used to assess HRQoL in patients with acromegaly, thereby contributing to more standardized and patient-centered evaluations of disease impact.

## Methods

### Scoping review

This is a systematic scoping review of the literature on the tools available to assess QoL in people with acromegaly.

The scoping review method was selected as it suits the broad question of assessing the different tools used to evaluate QoL in people with acromegaly, allowing for various types of research to be summarized [27]. The scoping review adhered to the methodology outlined in the Joanna Briggs Institute Reviewer’s Manual [28] for conducting methodological scoping reviews. Reporting of the review follows the guidelines outlined in the Preferred Reporting Items for Systematic Reviews and Meta-Analyses extension for Scoping Reviews (PRISMA-ScR) [29].

### Research question

This study is guided by the following research question: *What disease-specific and generic instruments have been used to assess HRQoL in people with acromegaly?*

### Inclusion criteria

*Type of participants:* participants were adults with acromegaly. Disease control and type of treatment, surgery, radiotherapy, or medications were not regarded as exclusion criteria.

*Concept:* Quality of life questionnaries for patients with acromegaly were identified and reviewed. No language restriction was applied. We assessed the questionaries following the review criteria proposed by the Scientific Advisory Committee of the Medical Outcomes Trust criteria [30]. The instruments were divided into generic and disease specific. We summarized the country of origin, focus, design, domains, number of items, mode of administration, time required for administration, and frequency of use. The psychometric properties of reliability, validity, and sensitivity to change for each instrument were evaluated following the methodology proposed by Solans and collaborators [31]. First, reliability was assessed by measuring internal consistency and test-retest reliability. Next, validity included structural validity, construct validity, and criterion validity. Finally, sensitivity to change was assessed using the effect size, considering a minimum effect size of 0.2 to be acceptable.

*Context:* This review focused on adults with acromegaly, treated or not, regardless of the disease status or control with medication.

*Sources:* We considered epidemiological and experimental studies, including clinical trials with or without a control group, quasi-experiments, cohort studies, case-control studies, and cross-sectional studies. Case reports and narrative reviews were not considered in this review but were screened for reference lists.

## Search methods for study identification

### Electronic searches

The following databases were searched from inception to the specified date.

· MEDLINE (Pubmed) until September 2022

· Embase until September 2022

· CINAHL until September 2022

No sources of grey literature were searched, as they were unlikely to include additional validated QoL questionnaires meeting psychometric quality standards. However, to minimize the risk of missing potentially relevant instruments, studies in any language, country, and publication date were included, and reference lists from relevant published articles, including trials, systematic reviews, meta-analyses, and narrative reviews, were screened. The details of the search strategy are provided in Supplementary Table 1.

### Study selection

**Fig. 1:**
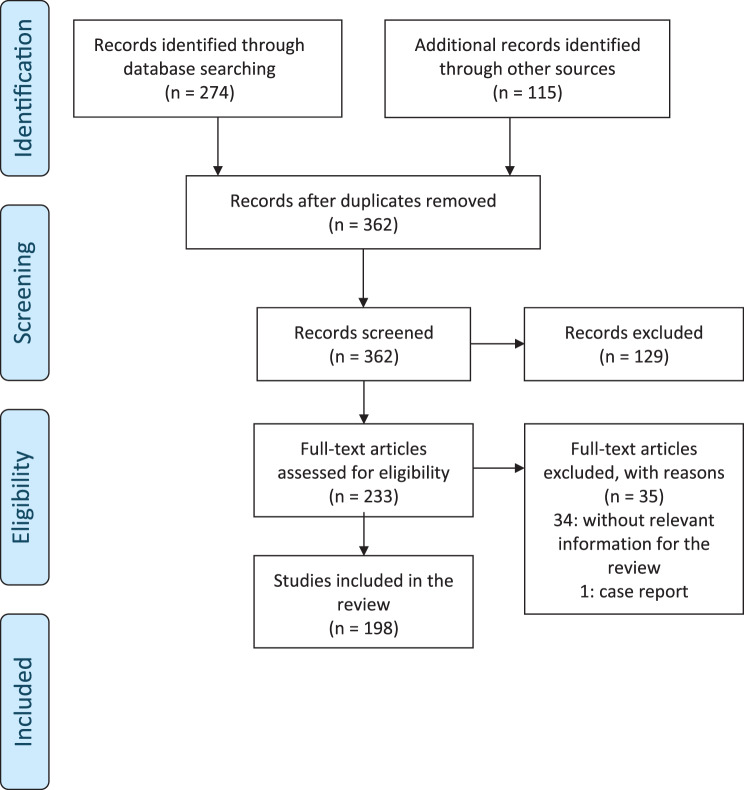
PRISMA flow diagram

Two authors (JECS, OFAR) independently screened all titles and abstracts. Discrepancies regarding eligibility—such as the type of study, population, or HRQoL focus—were first discussed to reach consensus. If disagreement persisted, a third author (CEBM) independently assessed the article and made the final decision, which was justified and recorded during group meetings. The selection process followed the recommendations in the PRISMA-ScR checklist [29], and a PRISMA flowchart of study selection is presented following the PRISMA statement (Fig. 1) [32].

### Data charting

Relevant information from each included study was captured in a data charting form that contains the following fields: Author(s), year of publication, country of origin, languages in which it is available, study population, design, domains, number of items, mode of administration, time of administration, the number of studies in which it has been used, transcultural validations, validity, reliability, and responsiveness to change.

### Presentation of the results

A narrative report was prepared to summarize the extracted data into generic and disease-specific QoL questionaries. Additionally, it was determined by consensus, following recommendations from [33], that the questionnaires assessing HRQoL should encompass more than one of the following aspects: physical health, psychological state, level of autonomy, and social relationships [33]. The instruments that met these requirements were classified as *multidomain*, and those that evaluated only one were referred to as *unidomain*. Finally, those that did not evaluate any of the proposed domains were labeled *with no domains*.

All the questionnaires were presented in a table of components that were assessed. Conceptual categories were selected via an iterative process. Finally, the authors provided a qualitative assessment of the questionnaires’ advantages and disadvantages, as defined by consensus.

### Classification framework for HRQoL instruments

**Fig. 2:**
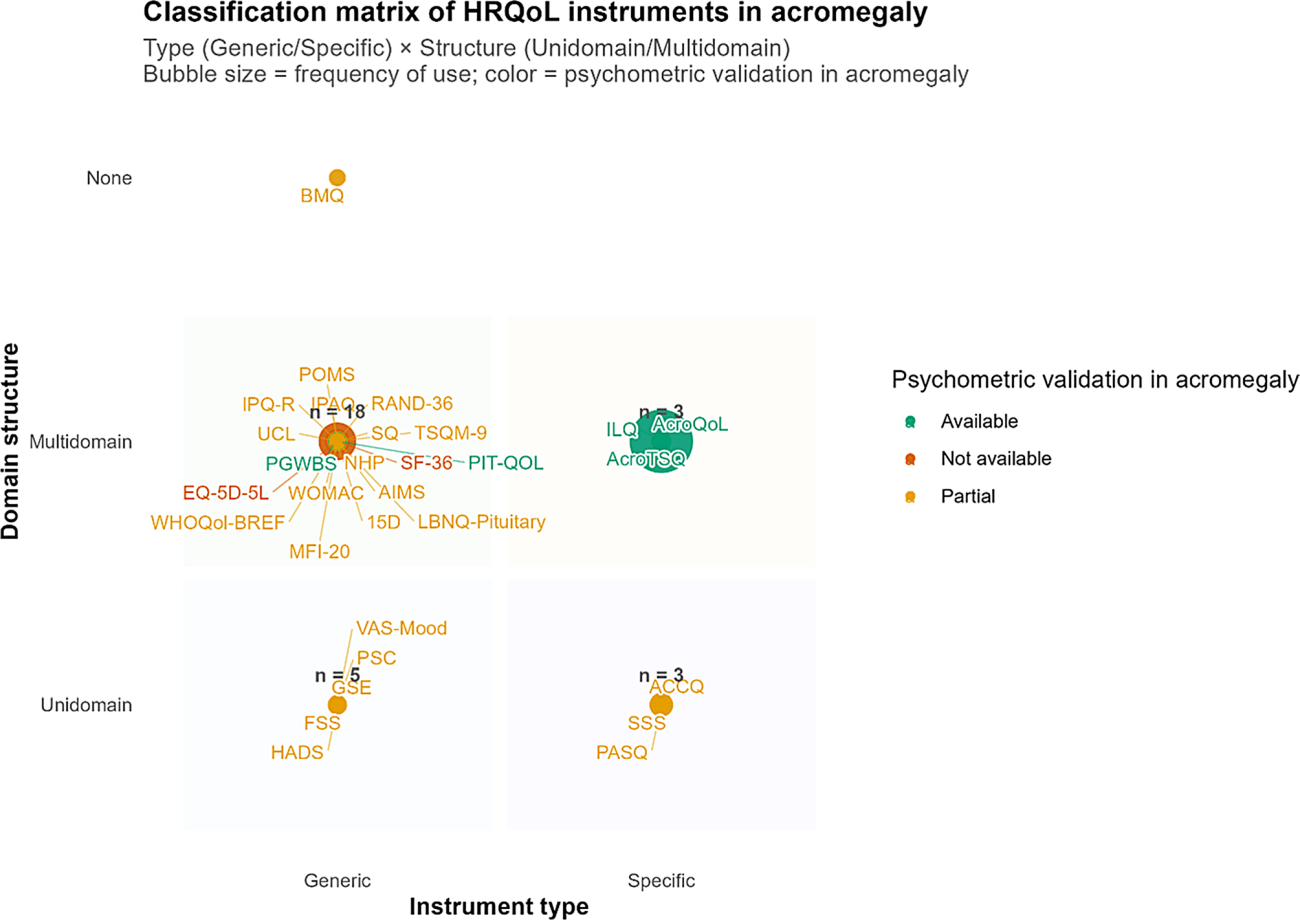
Classification matrix of HRQoL instruments used in acromegaly. Instruments are positioned according to their type (generic or disease-specific) and structural scope (multidomain or unidomain). Bubble size indicates the frequency of use across the included studies, and color represents whether psychometric validation has been reported specifically in acromegaly. Detailed instrument characteristics are summarized in Table 1, and categorical distributions in Table 2

To complement the descriptive synthesis, we developed a classification framework to organize the identified instruments according to two dimensions: [1] specificity—whether the instrument was disease-specific or generic—and [2] dimensionality—whether it assessed multiple or single domains of health-related quality of life (HRQoL). This framework, illustrated in Table 2 and Fig. 2, was defined a priori by the authors to facilitate interpretation and standardization of PROM use in acromegaly research.

The classification was informed by the conceptual model of HRQoL and by recommendations from the COnsensus-based Standards for the selection of health Measurement INstruments (COSMIN) initiative, which emphasize the importance of multidomain coverage when evaluating complex constructs such as quality of life [33]. Instruments were categorized as multidomain when they assessed more than one of the following core aspects: physical health, psychological state, level of autonomy, and social relationships. Questionnaires addressing only one of these aspects were classified as unidomain, and those not meeting either criterion were labeled non-domain.

**Fig. 3:**
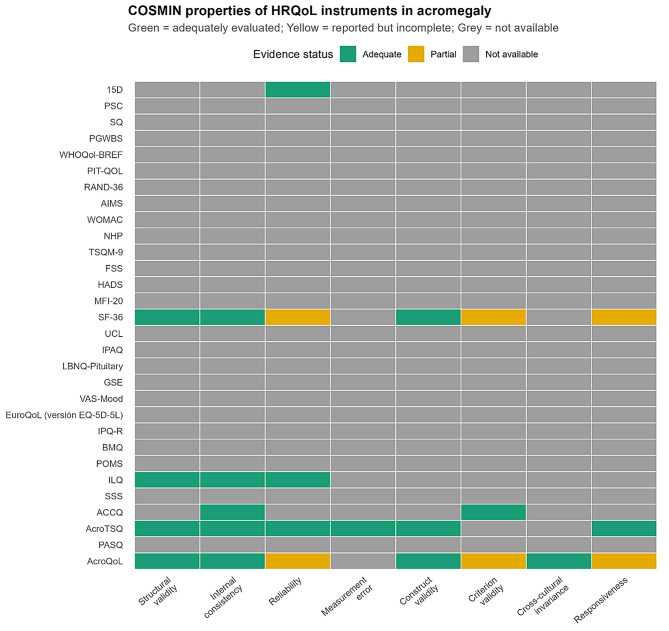
COSMIN heatmap of measurement properties by instrument. Cells summarize availability/quality of evidence for structural validity, internal consistency, test–retest reliability, measurement error, construct and criterion validity, cross-cultural validity/invariance, and responsiveness. Green = adequately evaluated; yellow = partial/insufficient reporting; grey = not available. Detailed sources are provided in Table 1

This conceptual grouping allowed the mapping of PROMs beyond their psychometric characteristics, highlighting their scope and clinical applicability. It also served as the basis for visual representations (Figs. 2 and 3), which synthesize the distribution of instruments by type, domain coverage, and psychometric evaluation status.

## Results

We identified 362 records across databases and other sources; after removing duplicates and screening, 198 articles were retained (Fig. 1). Across these, we found 30 questionnaires used to evaluate HRQoL in acromegaly, spanning disease-specific and generic tools and multidomain and unidomain structures, with their psychometric attributes summarized in Table 1 and a visual overview provided in Fig. 2 (classification matrix) and Fig. 3 (COSMIN heatmap).

**Table 1:** Summary of the questionnaires

Scale	First author of the original study	Year	Country of origin	Study population	Domains or subscales	Number of items	Mode of administration	Time to administer (minutes)	Frequency*	Structural validity	Internal consistency	Reliability	Measurement error	Hypotheses testing for construct validity	Cross-cultural validity\measurement invariance	Criterion validity	Responsiveness
Acro-QoL	Webb SM [21]	2001	Spain	Acromegaly patients	22:8 physical and 14 psychological	22	SR or AE	5	77	Original study: (-). Iranian patients: Through CFA [34]	Original study: Through Cronbach’s alpha (>0.9 for each scale and subscale)	Original study: (-). Iranian patients [34]: ICC = 0.907 for the questionnaire, 0.887, and 0.885 for the physical and psychological subscales, respectively.	(-)	(-)	Original study: (-). Iranian acromegaly patients [34]: (?)	Original study: (-). In the Japanese version [35], through correlation with SF-36	Original study: (-). In another study [17] through SES: global score −0.775, Physical −0.775, Body image −0.643, Personal relationships −0.428
PASQ	Trainer PJ [36]	2000	United Kingdom	Acromegaly patients	Does not apply	7	AE	15	12	(-)	(-)	(-)	(-)	(-)	(-)	(-)	(-)
Acro-TSQ	Fleseriu M [37]	2019	United States, United Kingdom, and Netherlands	Acromegaly patients	6: Symptom interference, gastrointestinal side effect interference, injection site interference, satisfaction with treatment, emotional reaction with treatment, and convenience of treatment	25	SR or AE	60	6	Original study: (-)	Original study: (-). In a multicenter study [39]: (-).	Original study: (-).	Original study: (-).	(-)	Original study: (-).	Original study: (-). In a multicenter study [39] through Pearson correlations: Symptom interference: Pearson correlations > 0.30 with the total score of the AIS instrument, with the “Alteration of activity” of the WPAI: SHP instrument, and with all EQ-5D-5 L scores at baseline, baseline and week 26.	Original study: (-). In a multicenter study [39] through SES, SRM, and RS
										Patients from Turkey [38] through CFA	In patients from Turkey [38] through Cronbach’s alpha (0.87)	In a multicenter study [39]: Through ICC: > 0.70 [range between 0.72 and 0.97]).	In a multicenter study [39]: via MDC (9–13 for Symptom Interference, 8–11 for Convenience of Treatment, 10 for Injection Site Interference, 7–12 for Gastrointestinal Interference, 7–10		In patients from Turkey [38]: (?)	- Convenience of treatment and Interference with Injection Sites: correlations > 0.30 with the “Activity alteration” component” of the WPAI: SHP instrument and with all EQ-5D-5 L scores between determinations, but the correlations were lower with the AIS instrument scores.	
													for Treatment Satisfaction, and 11–12 for Emotional Reaction) and MID (10–12 for Symptom Interference, 9–11 for Treatment Convenience and 8–10 for Gastrointestinal Interference)				
																- GI Interference and Satisfaction with Treatment exhibited correlations > 0.30 with the AIS total scores, the item “Activity Alteration of the WPAI: SHP and most EQ-5D-5 L scores at screening, baseline, and week 26. -Emotional reaction: correlations > 0.30 with the Anxiety/Depression item of the EQ-5D-5 L between the three determination points.	
ACCQ	Psaras T [40]	2011	Germany	Acromegaly patients	Eight components (not classified as domains): acral growth, joint discomfort, increased sweating, hypertension, paresthesias in fingers, sleep apnea, excess growth of facial features, and diabetes	8	SR	(-)	1	(-)	Original study: Through Cronbach’s alpha (0.709)	Original study: (?)	(-)	(-)	(-)	Original: Through Pearson correlations (correlated with the “General Health” Subscale of the SF-36 scale [Pearson correlation coefficient: *r* = 0.717, *p* < 0.01] and with the total score of the AcroQoL scale [Coefficient Pearson correlation: *r* = −0.585, *p* < 0.001])	(-)
SSS	Rowles SV [41]	2005	United States	Acromegaly patients	5: headache, sweating, joint pain, fatigue, and soft tissue inflammation [41]	5	SR or AE	5	2	(-)	(-)	(-)	(-)	(-)	(-)	(-)	(-)
ILQ	Lenderking WR [42]	2000	United States	Acromegaly patients treated with octreotide [42]	3: burden, lifestyle disruption, and compliance	42	SR or AE	(-)	1	Original study: (-). In patients in the United States, through FA [42]	Original study: Cronbach’s alpha for Compliance 0.86, for Burden 0.82 and Disruption 0.89	Original study: ICC for the three subscales (r 5.80, r 5.89 and r 5.89)	(-)	(-)	(-)	(-)	(-)
POMS	Mc Nair [43]	1971	United States	A wide range of populations, including healthy subjects and patients with variable medical conditions	Six components in 6 subscales: tension-anxiety (Pten), depression-dejection (Pdep), anger-hostility (Pang), confusion-bewilderment (Pcon), fatigue-inertia (Pfat), and vigor-activity (Pvig)	30	SR	5–7	2	Original study: (-). In postmenopausal women [44]: Through RMSEA (0.078) and CFI (0.97)	Original study: (-). In postmenopausal women [44]: Through Cronbach’s alpha (0.84 and 0.94)	Original study: (?). The fatigue subscale was evaluated in a survey of young Korean patients [45]: 0.66.	(-)	(-)	Original study: (-). Evaluated in other studies [46]: (?)	(-)	Original study: (-). In postmenopausal women [44]: ?.
BMQ	Horne R [47]	1999	United Kingdom	The general population who receive some medication	There are two sections (BMQ-General and BMQ-specific), each divided into two subscales. -BMQ-General section: 8 items divided into two subscales: 1) Overuse subscale: evaluates beliefs that medications are overprescribed.	18	AE	15	1	Original study [47]: Through the CFA (for the BMQ-General: Overuse subscale = 0.70, Harm subscale = 0.73. For the BMQ-Specific: Needs subscale = 0.95, Concerns subscale = 0.90	Original study: Through Cronbach’s alpha (general medicine patients:	The original study [47], through ICC, only reported on asthmatic patients: -BMQ-General-Subscale Overuse: 0.60	(-)	(-)	Original study: (-). In patients from Hungary, Slovakia, Czech Republic [48]: (?)	Original study: (-). In women with breast cancer [49], correlation with MARS scale through Spearman’s R	(-)
					2) Harm subscale: evaluates beliefs about medications as harmful, addictive, or poisonous. -BMQ-Specific Section: 10 items divided into two subscales: 1) Need subscale: focuses on the perceived need to take medications to stay healthy 2) Worries subscale: focuses on concerns about the adverse effects of taking medications [47]						BMG-General-Overuse Subscale = 0.60, Harm Subscale = 0.51. BMQ-Specific-Needs subscale = 0.86, Concerns subscale = 0.65)	-BMQ-General-Subscale Damage: 0.78 -BMQ-Specific-Needs Subscale: 0.77 -BMQ-Specific-Concerns Subscale: 0.76					
IPQ-R	Moss-Morris R [50]	2004	United Kingdom and New Zealand	Patients with chronic diseases: asthma, diabetes, rheumatoid arthritis, chronic pain, acute pain, myocardial infarction, multiple sclerosis, and HIV	3: Illness identity (14 items), Illness perception (28 items), and Causal attributions (18 items) [51]. Other authors have divided it into nine subscales: Identity, Causes, Temporality (cyclical), Temporality (Acute/chronic), Consequences, Personal control, Treatment control, Coherence of the disease, and Emotional representations [52]	70	AE	10–15	3	Original study: (-). In patients with prostate, breast, and colorectal cancer [53]: Through CFI (0.879), RMSEA (0.060 [0.056–0.063]), and SRMS (0.064)	Original study: Through Cronbach’s alpha (0.79 for the cyclical dimension of the timeline and 0.89 for the acute/chronic dimension of the timeline).	Original study: (-). In the Chinese version, it was applied in women with urinary incontinence [54] through ICC (0.80 and 0.94)	(-)	(-)	Original study: (-). German version [55]: (?)	Original study: (-). In other studies [56, 57] through Spearman correlations	(-)
EuroQoL (versión EQ-5D-5 L)	EuroQoL Group [58]	1990	Finland, Netherlands, Norway, Sweden, and the United Kingdom [59]	General population and in patients with different pathologies [60]	5: mobility, self-care, usual activities, pain/discomfort, and anxiety/depression	5	SR or AE	5	13	Original study: (-). In patients with depression [61]: Through CFA and RMSEA	Original study: (-). In patients with spondyloarthritis [62]: Through Cronbach’s alpha (0.79)	Original study: (-). In patients with depression [61]: Through Cronbach’s alpha	(-)	(-)	Original study: (-). In another study [63] through the DIF	Original study: (-). In patients with depression [61]: Through correlation with the PHQ-9 and ED-VAS questionnaire	Original study: (-). In patients with depression [61]: via SES and SRM
VAS-Mood	Folstein MF [64]	1973	United States	The study by Folstein et al. was applied in 2 samples: sample A) Male patients from the armed forces hospitalized in psychiatric and orthopedic areas of a United States Naval hospital. Sample B) Psychiatric inpatients at The New York Hospital, Westchester Division, a private psychiatric hospital	1: Mood	1	SR or AE	(-)	1	(-)	Original study: (-). In patients with irritable bowel syndrome [65] through Cronbach’s alpha (0.85)	Original study [64]: ?. Through the intragroup reliability coefficient (Sample A = 0.61 and Sample B 0.73) and the within-patient coefficient (Sample A = 0.32 and Sample B = 0.48)	(-)	(-)	(-)	Original study: (-). In patients with musculoskeletal pain [66], Spearman correlations with the SF-36 scale	Original study: (-). In patients with infertile dysfunction [67] evaluated through SRM
GSE	Schwarzer [68]	1979	Alemania	The general adult population, including adolescents	1: self-efficacy	20	SR	2–3	1	Original study: (-). In other studies [69]: Through RMSEA (≤0.08) and [70] RMSEA (0.09)	Original study: (-). In a study [69] through Cronbach’s alpha (0.86). Brazilian public officials [70] through Cronbach’s alpha (0.80).	Original study: (-). In Brazilian public officials [70] through the ICC (0.71 [0.66–0.77])	(-)	(-)	Original study: (-). In patients from Uganda [71] through DIF	Original study: (-). In Ugandan patients [71]: Through Pearson correlations	(-)
LBNQ-Pituitary	Andela CD [19]	2016	Netherlands	Patients with pituitary diseases	5: mood, negative perceptions of illness, problems in sexual functioning, physical and cognitive complaints, problems in social functioning	26	AE	(-)	2	(-)	Original study: Through Cronbach’s alpha (>0.765 for each factor)	(-)	(-)	(-)	(-)	Original study: correlation with the scales EQ-5D (−0.599 to 0.534), SF-36 (−0.690 to −0.220), MFI-20 (−0.265), HADS (0.612–0.716), CushingQol (−0.884) and AcroQoL (−0.661 to −0.563)	(-)
IPAQ	Cardol M [72]	1999	Netherlands	People with chronic diseases	5: family role, autonomy abroad, autonomy inside, life and social relationships, work and education	32	SR or AE	30	1	Original study: (-). In patients with multiple sclerosis [73] through CFA	Original study: (-). In a study of patients in rehabilitation centers [74] through Cronbach’s alpha (0.91 for internal autonomy, 0.90 for family role, 0.81 for external autonomy, 0.86 for social relationships, and 0.91 for job opportunities and educational)	Original study: (-) In patients in rehabilitation centers [74]: Through the ICC (Autonomy inside = 0.87, family role = 0.83, autonomy outside = 0.91, and social relationships = 0.89)	(-)	(-)	Original study: (-). In patients from Germany and England [75]: Through the DIF.	Original study: (-). In the Thai version [76], evaluated through Spearman correlations	Original study: (-). In patients in a rehabilitation department [77] through SRM (participation domain ranged between 0.1 and 1.3, problem experience item ranged between 0.4 and 1.5) and AUC (participation domain ranged between 50 and 92%, problem experience item ranged between 56 and 74%)
UCL	Scheurs P [78]	1993	Germany	General population	7: active coping, palliative coping, passive coping, avoidant coping, seeking social support, expressing emotions, and calming thoughts	47	SR or AE	(-)	1	(-)	Original study: (-). English version in UK patients [79]: Through Cronbach’s alpha (0.64 - 0.82)	Original study: (-) In UK patients [79]: (?)	(-)	(-)	(-)	Original study: (-). In patients from the United Kingdom [79], it was compared with the COPE (Coping Orientation to Problems Experienced) scale: Spearman’s r in men between 0.24 and 0.71; in women between 0.42 and 0.76	(-)
SF-36	Ware J [80]	1992	United States	Designed for use in clinical practice, health policy evaluations, and general population surveys.	9: physical functioning, social functioning, limitation of functions (physical), limitation of functions (emotional), mental health, vitality, pain, general perception of health, general perception of changes in health.	35	SR or AE	7	35	Original study: (-). In patients with chronic pain [81]: Through RMSEA (0.041 [90% CI: 0.041–0.042]), SRMSR (0.038), TLI (0.971), and CFI (0.976)	Original study: (-). In elderly patients in the community [82]: Through Cronbach’s alpha (0.80)	Original study: (-). In community elderly [82]: Through ICC (0.83)	(-)	(-)	Original study: (-). In the Danish version [83] through DIF	Original study: (-). In patients with sick sinus syndrome [84]: (?), through CR and AVE	Original study: (-). In patients with spinal surgical procedures [85], through SRM and SES
MFI-20	Smets EM [86]	1995	Netherlands	Cancer patients, chronic fatigue syndrome patients, psychology students, medical students, military personnel, and doctors	5: general fatigue, physical fatigue, mental fatigue, reduced activity, and reduced motivation	20	SR	5–10	6	Original study: Through the CFA (0.96 and 0.98).	Original study: Through Cronbach’s alpha (average of 0.84)	Original study: (-). In the general population [87] through ICC	(-)	(-)	Original study: (-). In patients with post-polio syndrome [88] through DIF	Original study: correlation with a visual analog scale that measures fatigue: (?). In a study of German patients [89], it was correlated with SF-36 through Spearman correlations (0.71–0.85).	Original study: (-). In Norwegian patients with inflammatory bowel disease [90]
HADS	Zigmond AS [91]	1983	England	Patients in an outpatient clinic	2: anxiety and depressive symptoms	14	SR	2–5	5	Original study: (-). In a study of Australian community patients [92] through CFA	Original study: (-). In German patients [93]: Through Cronbach’s alpha (average of 0.80)	Original study: (-). In German patients [93] through the ICC (0.89, 0.86, and 0.91 between the scores of the anxiety and depression subscales and total scores).	(-)	(-)	Original study: (-). In Nigerian patients with chronic low back pain [94]: (?)	Original study: (-). In Colombian patients [95] through Spearman’s R (0.65) when compared with scores from the PHQ-9 questionnaire	Original study: (-). In patients with multiple sclerosis [96] and patients with COPD [97]: (?)
FSS	Krupp L [98]	1989	United States	The original study was applied to patients with multiple sclerosis and systemic lupus erythematosus	1: fatigue	9	SR	(-)	1	Original study: (-). The Greek version was evaluated in a non-acromegalic population [99] through CFA, CFI, and SRMR.	Original study: Through Cronbach’s alpha (in healthy adults = 0.88, in patients with lupus = 0.89 and in patients with multiple sclerosis = 0.81)	Original study: Through the ICC (in healthy adults = 0.20, patients with multiple sclerosis = 0.26, and patients with lupus = 0.46).	(-)	(-)	Original study: (-). In the Portuguese version in non-acromegalic patients [100]: (?)	Original study: (-). In patients with post-stroke fatigue [101]: correlation with Fatigue domain of the VAS questionnaire and with SF-36 through Spearman correlations (all = r > 0.60)	Original study: (-). In obese patients [102]: Through SRM (=0.50)
TSQM-9	Atkinson M(2004 original Version) [103] - Bharmal M (2009 Version) [104]	2004	United States	2004 Version: chronic pathologies: arthritis, asthma, major depression, type 1 diabetes, dyslipidemia, hypertension, migraine, and psoriasis [103]. Version 2009 Patients receiving antihypertensive medication [104]	2004 version effectiveness, side effects, convenience and overall satisfaction [103] 2009 version without the side effects domain [104]	14	SR or AE	(-)	1	Original study: evaluated through CFA [104]	The original study [104] and other studies [103]: Through Cronbach’s alpha	The original study [104] and other studies [103] evaluated through an intraclass correlation coefficient (greater than 0.70)	(-)	(-)	(-)	Correlation of the modified Morisky scale score [103, 104]	(-)
NHP	Hunt SM [105]	1976	London	Specific groups of patients or the general population. Validation: osteoarthritis and peripheral vascular disease [106]	The first part’s six categories are sleep, physical mobility, energy, pain, emotional reactions, and social isolation. The second part 7 categories: employment, domestic activities, social life, home life, sexual life, hobbies and interests, and vacations [106, 107]	45	SR or AE	5	4	(-)	(-)	Original study: (-). In validation study: high test-re-test, correlation coefficient indices [106]	(-)	(-)	(-)	(-)	(-)
WOMAC	Bellamy M [108]	1982	Canada	Patients with hip and knee osteoarthritis, rheumatoid arthritis, juvenile rheumatoid arthritis, fibromyalgia, systemic lupus erythematosus, and low back pain [109]	3: pain, stiffness, and physical function TTT	24	SR or AE	12	1	Original study: (-). Validation study: agreement coefficient called Lin’s correlation [109]	Original study: (-). Validation study: Cronbach’s alpha (0.86 Likert version) - (0.89 VAS version) [109]	Original study: (-). Validation study: test-retest - ICC [109]	(-)	(-)	(-)	(-)	Original study: (-). Validation study: SES and SRM [109]
AIMS	Meenan RF et al. [110]	1980	United States	Patients with arthritis and osteoarthritis	9: mobility, physical activity, social activity, social role, home activities, pain, dexterity, anxiety, and depression [110]	45	SR or AE	15	1	(-)	Original study: Cronbach’s alpha [110]	Original study: test-retest: ICC is more significant than 0.70. [110]	(-)	(-)	(-)	(-)	(-)
RAND-36	Hays RD [111]	1993	United States	General population. In the original: English-speaking adults with one or more chronic diseases: high blood pressure, diabetes, heart disease, or depression [111]	Eight subscales: physical functioning, role limitations caused by physical health problems, role limitations caused by emotional problems, energy/fatigue, emotional well-being, social functioning, pain 2, general health perceptions [112]	36	SR or AE	10	2	Original study: (-). Brazilian version: CFA, CFI and TLI > 0.9 [113]	Original study: (-). Swedish version: Cronbach’s alpha (>0.80) [114]	Original study: (-). Interclass correlations (between 0.71 and 0.93) Brazilian version (between 0.71 and 0.94) [113]	Original study: (-). Brazilian Version: RMSEA [113]	(-)	(-)	Original study: (-). Brazilian version: The convergent validity of RAND-36 showed an excellent correlation for all domains, except the domains of Pain and Social Functioning with medium correlations [113]	(-)
PIT-QOL	Kan P [115]	2006	Canada	Patients with pituitary adenoma [115]	6: General health, Emotional health, Social/family well-being, Health problems related to a pituitary tumor, Treatment of pituitary tumor, Relationship with the doctor [115]	54	SR or AE	10	1	(-)	(-)	Original study: test-retest (first score vs. second 21.42 vs. 20.68, *p* = 0.73), with a Pearson correlation coefficient of 0.88 [115]	(-)	(-)	(-)	Original study: Through Pearson correlation coefficients = 0.75, compared to RAND-36 [115]	(-)
WHOQol-BREF	WHOQOL Group [116]	1998	18 Countries, including Latin America	General population	4: Physical, Psychological Health, Social Relationships, Environment [116]	24	SR or AE	(-)	3	Original study: (-). In the Norwegian general population [117]: Satisfactory FA, fit index: 0 ± 90, CFI 0 ± 900	Original study: (-). In other populations: Cronbach’s alpha with good internal consistency. [117, 118]	Original study: (-). In other studies: high correlation (0 ± 56 to 0 ± 84) [117, 118]	(-)	(-)	(-)	(-)	(-)
PGWBS	Wenger NK [119] - Short version [120]	1984 - Short version 2006	United States	General population	6: Anxiety, depression, positive well-being, self-control, general health, vitality [41]	Original: 22. Short version: 6	SR or AE	(-)	3	(-)	In the original study: (-). In other articles, Cronbach’s alpha for both versions = 0.82 to 0.94 [120, 121]	Original study: (-). In other articles: test-retest coefficients = 0.80 [121]	(-)	(-)	Original study: Cross-cultural validation [120] (?)	(-)	(-)
SQ	Kellner R [122]	1976	United States	Psychiatric patients (original study) and the general population	Two domains: Psychological (Depression, anxiety, hostility, and somatization) and Well-being (satisfaction, relaxation, kindness, and physical well-being) [123]	92	SR or AE	(-)	1	(-)	(-)	Original study: (-). In other studies: ICC (0.71 [0.66–0.77]) [124]	(-)	(-)	(-)	Original study: (-). In other studies [123], ICC ranged between 0.39 and 0.93 compared to other scales	(-)
PSC	DSM-III Diagnostic and statistical manual of mental disorders [125]	1980	Estados Unidos	General population	General/neurological, autonomic, musculoskeletal/pain, gastrointestinal, genital, hot/cold sensation. The presence of symptoms is classified on a severity scale from 0 to 3. [125, 126]	55	SR or AE	(-)	2	(-)	(-)	(-)	(-)	(-)	(-)	(-)	(-)
15D	Sintonen H [127]	1970	Finland	General population - Elderly and frail people	15: Breathing, mental function, communication, vision, mobility, habitual activities, vitality, hearing, feeding, elimination, sleep, distress, discomfort and symptoms, sexual activity, and depression. Each dimension is divided into five levels [128]	15	SR or AE	5–10	1	(-)	(-)	Original study: high test-retest coefficients [128]. In other studies, test-retest repeatability coefficients were high in patients awaiting bypass surgery [128]- Portuguese version test-retest were between 0.77 and 0.97 [129]	(-)	(-)	Original study: (-). Portuguese version [129] (?) Greek version [130]: (?)	Original study: (-). In other studies, correlation was −0.64 with SF-20 and HDRS [131]	(-)

(-): Information not available or insufficient. (?): reported but not according to COSMIN guideline criteria. AE = Administered by Evaluator. AVE = Average Variance Extracted. CFA = Confirmatory Factor Analysis. CFI = Comparative Fit Index. CR = Composite Reliability. DIF = Differential Item Functioning. ES = Effect Size. FA = Factor Analysis. G* = Generic. ICC = Intraclass Correlation Coefficient. MDC = Minimal Detectable Change. MID = Minimally Important Difference. RMSEA = Root Mean Square Error of Approximation. RS = Responsiveness Statistic. S* = Specific. SES = Standardized Effect Size. SR = Self-Reported. SRM = Standardized Response Mean. SRMR = Standardized root mean residuals. TLI = Tucker–Lewis Index

*The number of studies that had reported the use of the scale

To facilitate navigation, Table 2 synthesizes the classification by instrument type and domains, Fig. 2 locates each PROM in a 2 × 2 matrix (bubble size = frequency of use; color = psychometric evaluation in acromegaly), and Fig. 3 displays a COSMIN heatmap of measurement properties. Detailed instrument-level descriptions remain available in Table 1.

**Table 2:**
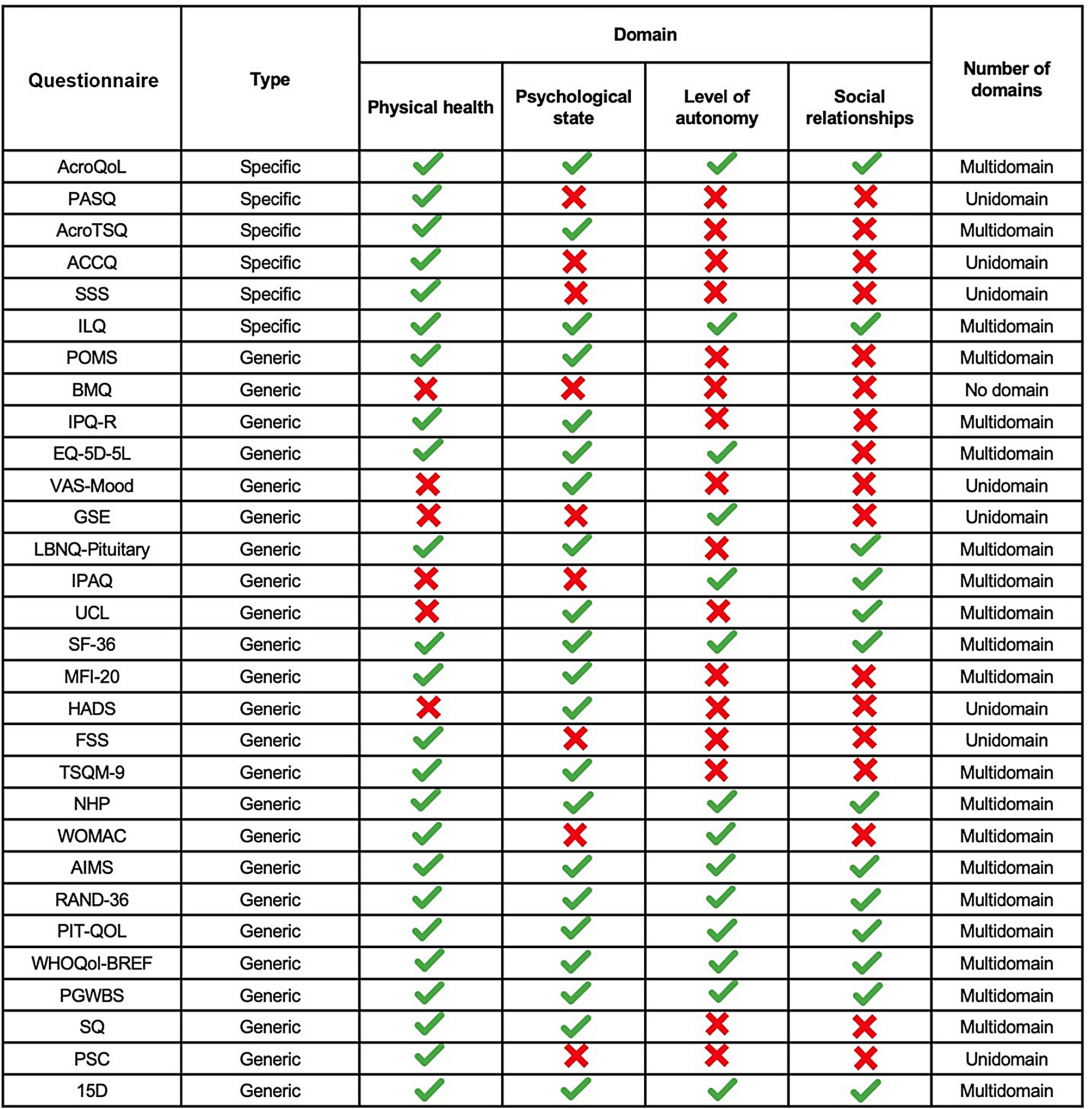
Classification of instruments according to type and number of domains evaluated

Several instruments remain underused in acromegaly despite their broader application in other clinical contexts. Common reasons include their unidimensional scope (e.g., symptom-specific scales that fail to capture broader HRQoL domains), administrative burden (e.g., administration times exceeding 10–15 minutes or requiring interviewer administration), lack of acromegaly-specific cross-cultural adaptation or validation, and clinicians’ preference for widely validated generic tools that enable cross-disease and economic comparisons. When used, these instruments are often applied within self-management interventions or in specific clinical subpopulations (e.g., targeting fatigue, mood, or treatment adherence), serving more as complementary measures rather than core HRQoL assessments. (See illustrative examples in Supplementary Table S1 and detailed psychometric properties in Table 1.)

### Questionnaires that assess HRQoL

Thirty questionnaires were used to evaluate HRQoL in acromegaly: 3 specific-multidomain, 18 generic-multidomain, 3 specific-domain, 5 generic-domain, and 1 that did not meet any of the proposal criteria.

The acromegaly-specific ones are Acromegaly Quality of Life (AcroQoL) [21] Patient Assessment Acromegaly Symptom Questionnaire (PASQ) [34], Acromegaly Treatment Satisfaction Questionnaire (AcroTSQ) [35], Acromegaly Comorbidities & Complaints Questionnaire (ACCQ) [36], Signs and Symptoms Score (SSS) [37] and the Impact on Lifestyle Questionnaire (ILQ) [38].

The generics were: Profile of Mood States (POMS) [39], Beliefs about Medicines Questionnaire (BMQ) [40], Illness Perception Questionnaire-Revised (IPQ-R) [41], EuroQoL-5D version EQ-5D-5 L [42], the “Mood” domain of the Visual Analogue Scale (VAS) [43], GSE (General self-efficacy scale) [44], Leither Bother and Needs Questionnaire for patients with Pituitary Diseases (LBNQ-Pituitary) [19], Impact on participation and autonomy (IPA) [45], Utrecht coping list (UCL) [46], Short Form-36 (SF-36) [47], Multidimensional fatigue inventory (MFI-20) [48], Hospital Anxiety and Depression Scale (HADS) [49], Fatigue Severe Scale (FSS) [50], Treatment Satisfaction Questionnaire for Medication (TSQM-9) [51], Nottingham Health Profile (NHP) [52], Western Ontario McMaster Universities Osteoarthritis Index (WOMAC) [53], Arthritis Impact Measurement Scale (AIMS) [54], The Rand 36-item health survey (RAND-36) [55], Pituitary Quality of Life Questionnaire PIT QOL [56], World Health Organization Quality of Life, short version (WHOQOL-BREF) [57], Psychological General Well Being Scale (PGWBS) [58], Kellner’s Symptom Questionnaire (SQ) [59], Physical Symptoms Checklist (PSC) [60], 15 D instrument of health-related quality of life (15 D) [61]. Table 1 provides a comprehensive synthesis of the psychometric properties of each instrument, while Table 2 presents a classification according to the type of questionnaire (generic or specific) and the number of domains.

### Specific-multidomain questionnaires

AcroQoL is a specialized instrument designed to assess HRQoL in people with acromegaly. It considers physical and psychological domains, with the latter divided into two dimensions: physical aspect and personal relations [21]. Originally developed in Spanish, it is currently available in other languages [62, 63] and has been validated using generic scales [17], making it the most validated specific acromegaly scale. The instrument has been evaluated across multiple settings including the general acromegaly population [18, 64–75]; postmenopausal women [76]; patients undergoing surgical treatment [77] or medical treatment [63, 78–83]; people with acromegaly affected by gastrointestinal [84] and neurological conditions [85] people with vertebral fractures [86] or arthropathy [87]; in men with erectile dysfunction [88]; and in patients undergoing rehabilitation therapies [89, 90] among other multiple scenarios. It has also been applied to evaluate patient perceptions of the disease and medical treatment [91, 92], the usefulness of intervention programs for self-care [132], patients’ functional capability [133, 134], the influence of work aspects on quality of life [135], and for the development of other acromegaly questionnaires [19].

Acro-TSQ is a PROM designed for implementation in people with acromegaly who receive treatment with somatostatin analogs [35]. It contains 27 items, but a variation has been proposed [93]. It was designed to assess the burden of the disease and the patient satisfaction with the treatment, allowing its use in clinical trials [94–97].

The Impact on Lifestyle Questionnaire (ILQ) integrates the SF-12, which evaluates the physical and mental health domains, with 30 additional items specifically developed to capture the lifestyle impact of octreotide treatment. These items were derived from a literature review of studies examining treatment burden and patient-reported quality of life [38, 98]. The instrument assesses multiple aspects, including treatment burden, lifestyle disruption, adherence, satisfaction with therapy, and patients’ perceptions of treatment impact and effectiveness. It comprises three subscales, compliance, burden, and lifestyle disruption—that together provide a comprehensive evaluation of the treatment’s effect on daily living [38].

### Generic-multidomain questionnaires

POMS. Initially introduced in 1971 by McNair et al. [39], POMS assesses mood disorders on a response scale of 0 to 4 for each item, with higher scores indicating increased symptomatology experienced over the past week. Psychometric properties, as detailed in Table 1, have undergone validation against various questionnaires, including the Greene Climacteric Scale in postmenopausal women [99], and against scales used in cancer patients, such as the Center for Epidemiologic Studies-depression (CES-D), Medical Outcomes Study (MOS) in its abbreviated 20-item form, Self-Rated Karnofsky Performance (SR-KPS), and Bradburn Positive and Negative Affect [100]. Anagnostics et al. [136] employed the Greek version of this instrument in individuals diagnosed with Acromegaly. The instrument has also been used in studies on acromegaly patients [101] not focusing quality of life.

IPQ-R. This is a revised version of the Illness Perception Questionnaire (IPQ), developed by Moss-Morros et al. in 2002 [50]. The instrument evaluates components of the representation of the illness according to Leventhal’s Common Sense Model and addresses perceptions of the disease [102–105]. The IPQ-R is structured into three components: illness identity, perception, and causal attributions [91]. Some researchers have applied it in studies involving patients with acromegaly to evaluate outcomes such as beliefs about medication, perceptions of the disease, and quality of life [91, 106, 107]

EQ-5D. A group of instruments created by the EuroQoL group that describe and evaluate HRQoL reflected in five dimensions: anxiety/depression, pain/discomfort, usual activities, self-care, and mobility. Each dimension receives a score of 1 to 3, with lower scores indicating a better quality of life. The instrument includes a visual analog scale (VAS) that rates current health-related well-being [108] with a possible score of between 0 and 100, with lower scores indicating a worse state of health. EQ-5D has two main versions, the EQ-5D-3 L and the EQ-5D-5 L, the latter created by the EuroqoL group to improve the sensitivity of the first version [109]. Both versions of EQ-5D have been evaluated in multiple population groups with different health conditions [109–115] (see Table 1). The questionnaire has also been applied in patients with acromegaly in the following scenarios: HRQoL in general [73, 116], QoL in patients who received any kind of treatment [18, 117], in those treated explicitly with somatostatin analogs [78], and in patients with biochemical remission [72]. It has also been employed to assess disease perception [91, 106, 107], develop intervention programs for self-care [118] implement other questionnaires for acromegaly [19], and compare with other questionnaires evaluating health status [37].

LBNQ-Pituitary. Developed by Andela et al. [19] the instrument is applied to patients with pituitary diseases to assess any negative effects induced by the disease, specifically the degree to which patients are bothered by their condition and their need for support from healthcare providers. Developed from focus groups, it covers five subscales: mood problems, negative illness perceptions, physical and cognitive complaints, sexual function issues, and social function issues. It is applied through interviews with patient groups, from which a possible score between 0 and 100 can be calculated. Higher scores indicate a higher level of perceived disturbance [19]. In individuals diagnosed with acromegaly, it was evaluated in conjunction with other questionnaires in a study that explored the benefits of a self-care intervention program [118] and another study designed to develop a disease-specific questionnaire [19].

IPA (IPAQ). This serves as a comprehensive assessment tool aimed at gauging the autonomy and participation of patients with chronic diseases, addressing limitations observed in other instruments measuring these dimensions. It consists of three main domains: activity, participation, and autonomy. The first explores the patient’s ability to perform physical, leisure, and daily activities. The second domain evaluates the individual’s ability to take part in social activities. The third and final segment explores the individual’s ability to make decisions and control their own life. Each question is answered on a five-point scale, from “I have no problems” to “I have serious problems.” At the end of the questionnaire, a score is calculated for each section, and an overall score indicates the overall impact of the disease on patient participation and autonomy [119]. The IPAQ is a valuable instrument for evaluating the effects of acromegaly on patients’ daily routines. Its application provides an effective way for healthcare professionals to effectively identify and address the daily limitations and challenges faced by individuals affected by acromegaly. It can also be used to evaluate the effectiveness of different treatments in improving the participation and autonomy of patients with acromegaly [119, 120]. In acromegaly, Andela et al. [118] applied it to assess the impact of a self-care intervention program along with other questionnaires.

UCL. Used to analyze coping strategies, the instrument comprises 47 items covering seven empirically derived subscales: active coping, seeking social support, palliative coping, avoidant coping, passive coping, fostering reassuring thoughts, and expressing emotions. For grading, each statement is assigned a score from 0 to 4 [121]. Limited information is available regarding the psychometric properties of this questionnaire [122] (see Table 1). Within the specific context of acromegaly, it has been incorporated alongside other instruments as an integral component of self-care intervention programs, emphasizing psychological facets [118].

SF-36. Introoduced in 1992 as part of the Medical Outcomes Study (MOS) [47] in the US, the instrument is designed to offer a comprehensive health status profile, and has proven effective for assessing HRQoL in the general population and specific subgroups [123]. The psychometric properties of the SF-36 have been examined in depth in numerous studies [124] across various countries and contexts [125, 126] (Table 1). It is one of the most commonly used generic questionnaires in studies related to acromegaly and includes evaluations of overall HRQoL [16, 67, 127–129] in patients undergoing various treatments such as general treatment [18, 130, 131], surgical treatment [20, 77, 137], medical treatment with Pegvisomant and somatostatin analogs [82, 136], and radiotherapy [138], in active patients [139], controlled patients [70, 72, 140] or cured patients [65]. The SF-36 has also been employed to assess various clinical conditions associated with acromegaly, such as vertebral fractures [86], gastrointestinal manifestations [141], musculoskeletal conditions [142–144], and oro-dental issues [145] as well as in patients with pituitary adenomas in general [120, 146] and in patients with growth hormone deficiency [147]. It has been used to evaluate the effects of self-care intervention programs [118] and assess perceptions of body image [148]. Finally, it has been applied in the context of occupational disability [117] and to evaluate gender differences in HRQoL [36].

MFI-20. Developed by Smets et al. to assess fatigue [48], the instrument comprises response scores ranging from 0 to 20, where higher scores indicate a higher level of fatigue [118]. It has been rigorously assessed in various contexts, including its application in evaluating HRQoL in acromegaly patients with long-term biochemical control [65], those with a history of prior radiotherapy [138], and individuals with osteoarthritis [144]. It has proven useful in delineating differences in HRQoL across age groups and genders [120], as well as in evaluating the effectiveness of self-care intervention programs [118], and as a reference for the development of other questionnaires applied to individuals with acromegaly [19].

TSQM-9. Derived from the original version 1.4 with 14 questions, this new version assesses effectiveness, potential side effects, convenience, and overall satisfaction, yielding scores for each of these four scales [149]. The TSQM-9 focuses on nine key items, excluding questions 4 to 8, which pertain to medication side effects. Scores within each domain of the TSQM-9 range from 0 (indicating lower satisfaction) to 100 (reflecting higher satisfaction) [51]. Psychometric properties are detailed in Table 1. This scale has been used to assess the efficacy of lanreotide autogel at 120 mg, administered at extended dosing intervals (> four weeks), demonstrating commendable levels of both satisfaction and treatment adherence [78].

NHP. Initially conceived to assess health and measure medical or social interventions, the NHP is frequently employed in patients with pituitary disease to evaluate overall well-being. It is divided into two parts: the first comprises 38 yes/no questions, subdivided into six categories (sleep, physical mobility, energy, pain, emotional reactions, and social isolation), reflecting the patient’s perception of the severity of the impact of their health. The second part consists of seven statements about health-related areas: paid employment, household chores, social life, personal relationships, sexual life, hobbies and interests, and vacations. Subscale scores are determined through a weighted average and presented on a scale of 0 to 100, with a higher score indicating lower quality of life [52, 150]. The questionnaire’s efficiency has been assessed in patients with acromegaly in remission and undergoing treatment with somatostatin analogs [65, 120]. It has also been used to assess acromegaly patients with biochemical control and also those receiving radiotherapy [138]. Clinical osteoarthritis has increasingly been recognized as a major determinant of quality of life, which has led to the application of the NHP in acromegaly patients presenting with osteoarthritic involvement of the spine, hip, and knee [144].

WOMAC. A self-administered form of 24 items utilized to assess hip and knee osteoarthritis distributed across three subscales: pain, stiffness, and physical function/functional capacity. Questions are rated on a scale of 0 to 4, where 0 represents ‘none,’ 1 ‘mild,’ 2 ‘moderate,’ 3 ‘severe,’ and 4 ‘extreme.’ The sum of scores from the three subscales provides a total score; a higher score indicates greater pain, stiffness, and functional limitations [53, 151].

AIMS is a self-assessment tool consisting of 45 items, encompassing nine subscales that evaluate aspects such as mobility, physical activity, agility, household tasks, social interaction, daily activities, pain, and levels of depression and anxiety [54]. AIMS and WOMAC have been used in patients with acromegaly to assess persistent complications affecting quality of life, such as motor disability and depression [75].

RAND-36. Consisting of 36 elements, following the same structure as the SF-36 described by Ware and Sherbourne, the instrument measures health status in any generic population and can estimate the overall quality of life in patients affected by pituitary tumors [152]. It includes eight domains: physical functioning, role limitations due to physical health problems, role limitations due to emotional issues, energy/fatigue, emotional well-being, social functioning, bodily pain, and general health [55, 153]. A single component reflects individual variation in the perception of personal health, with each scoring a maximum of 100. The total score reaches a maximum of 800, and scale scores are aggregated and converted to a scale of 0 (indicating the most unfavorable possible health status) to 100 (indicating the most favorable possible health status) [154]. This scale has been validated in Brazil and Sweden [154, 155], and its psychometric properties are described in Table 1. It has been used to determine the treatment’s effect on the course of health-related quality of life in acromegaly patients before and during the first 2.5 years of treatment [156]. It has also allowed for the measurement of quality of life in acromegalic patients after endoscopic transsphenoidal surgery, as well as in those who develop postoperative panhypopituitarism and are on adjuvant therapy (with somatostatin analogs) or receiving radiation [152].

PIT QOL. Designed for patients with pituitary adenomas, the PIT QOL comprises 54 questions categorized on a 7-point scale, where 1 denotes excellent quality of life, and 7 indicates poor quality of life. It is structured into six subscales addressing general health, emotional well-being, social interaction, health issues related to pituitary disease, treatment, and the relationship with the doctor. Higher scores reflect better quality of life [56]. It has been utilized in acromegalic patients following endoscopic transsphenoidal surgery [152].

WHOQOL-BREF. Derived from the WHOQOL-100, the instrument was developed in 20 centers in 18 countries, including Latin American nations such as Argentina, Brazil, and Panama. It comprises 26 questions distributed across four dimensions: physical health, psychological well-being, social relationships, and the environment. It assesses individual perceptions over the past two weeks [57]. Two of these questions focus on the general and separate assessment of quality of life and personal health [71]. Translated into 19 different languages, it applies to both healthy and ill individuals. Instead of focusing on functional aspects, it emphasizes the level of satisfaction the individual experiences in various everyday situations and is therefore considered the scale with the highest conceptual and methodological robustness [157]. Each item is evaluated on a scale of 1 to 5, and domain scores are obtained by multiplying the mean of all items within the domain by 4. This results in domain scores ranging from 4 to 20, where higher scores reflect a better quality of life [158]. Psychometric properties are described in Table 1. It has helped assess psychological factors in acromegaly patients and evaluate their relationship with quality of life in the context of disease control [69]. It has also been employed to assess quality of life in acromegaly patients compared to other chronic diseases such as pituitary adenomas, Cushing’s disease, psoriasis, and healthy individuals [159].

PGWBS. Initially described by Harold Dupuy in 1960 and subsequently revised, adapted, and validated in 1984 [58], the questionnaire consists of 22 items that assess affective states, with each item including six questions with responses on a scale of 0 to 5, referring to the past four weeks. It is divided into the following subscales: anxiety, depressed mood, positive well-being, self-control, general health, and vitality. The maximum score is 110, indicating excellent quality of life [37]. A short version with five items has been developed [160]. It has been adapted to many languages and cross-culturally validated in various countries [160]. In acromegaly patients, the PGWBS has been used to measure health-related quality of life by assessing overall psychological well-being and has been helpful in patients who received prior radiotherapy [37]. This questionnaire has been applied to determine changes in quality of life over time, the effect of different treatment modalities (184), and in patients with long-term biochemical remission [72].

SQ. A simple self-assessment questionnaire developed by Robert Kellner in 1976 [59], it was initially used to assess distress among neurotic patients participating in efficacy trials. It comprises domains related to psychological symptoms and well-being, with yes/no responses. The sum of the scores in the domains produces a total distress score [161]. Its psychometric properties have been described by the author [162] and are summarized in Table 1. The SQ has been translated into various languages, including Italian, French, German, Spanish, Portuguese, Dutch, Mandarin [161]. In acromegaly patients, it has been applied to determine the effects of lanreotide on quality of life [163].

15D. Designed to describe HRQoL, this instrument focuses on older individuals and other frail groups. It encompasses 15 dimensions, where the maximum score is 1 (indicating the absence of problems in all sizes), and the minimum score is 0 (equivalent to the condition of being deceased) [61]. It has been translated into different languages, such as Greek and Portuguese [132, 164] and has proven helpful in assessing the long-term HRQoL in patients with surgically treated pituitary adenomas, including patients with controlled and uncontrolled acromegaly [133].

### Specific-unidomain questionnaires

PASQ. Initially introduced in 2007 [36], this tool evaluates symptom severity in patients with acromegaly. Currently available in nine languages, including English, Italian, Danish, Spanish, Portuguese, Swedish, Dutch, German, and Slovenian, the PASQ has become one of the most widely used questionnaires for assessing clinical manifestations in patients with acromegaly [134]. PASQ has been employed in various studies [135]. The scoring methodology involves rating each item on the questionnaire on a scale of 0 to 8, where lower scores indicate the absence of symptoms, and higher scores indicate more severe symptoms. A final question addresses overall health status, using a scale of 0 to 10, with 0 representing optimal health and 10 representing the worst possible health. The total PASQ score is the sum of individual scores, with a maximum score of 40 points [34, 135]. The assessment of quality of life in patients with acromegaly using the PASQ has been the focus of numerous studies [76, 165, 166], some of which delve into specific aspects such as evaluation during treatment [167] or post-treatment with somatostatin analogs and Pegvisomant [34, 81, 135, 168–170]. It has also been applied in preoperative contexts [171] and patients with arthropathy [87].

ACCQ. A specific instrument designed to investigate the implications of signs, symptoms, and comorbidities caused by acromegaly on patients’ quality of life. It comprises eight items addressing complaints and comorbidities. Each item is assessed using a scoring scale of 0 to 3, reflecting the severity of symptoms (none, mild, moderate, severe). The maximum possible score is 24, where higher scores indicate greater impairment in quality of life. The interpretation of the results suggests that a score equal to or less than 8 implies that the patient experiences a low degree of discomfort due to the comorbidities; scores from 9 to 16 indicate mild to moderate pain, while a score equal to or greater than 17 indicates severe distress. The seminal study that birthed the instrument revealed that finger paresthesias emerged as the foremost contributor to the decline of quality of life. It also revealed nuanced distinctions in underlying factors which were observed between women and men. For women, the predominant influence stemmed from complications such as arterial hypertension, while for men, joint manifestations [37] played a more significant role in influencing their quality of life.

SSS. Derived from the Patient Acromegaly Symptom Questionnaire (PASQ), the SSS condenses five questions appraising symptoms such as headache, sweating, joint pain, fatigue, and soft tissue swelling on a scale of 0 to 8, offering a comprehensive evaluation of symptom severity. A maximum score of 40 signals the presence of severe signs and symptoms [37]. This questionnaire focuses on the more reversible aspects of acromegaly and has been used to assess patients with acromegaly in general [37] and in both controlled and uncontrolled patients [172].

### Generic-unidomain questionnaires

VAMS. The instrument has been applied in patients with acromegaly, notably in a study by Andela et al. [118], where it was used alongside other instruments to evaluate a self-care intervention program incorporating psychological components, with promising outcomes. Although the specific version of the instrument used in that study was not specified, the original VAMS developed by Folstein et al. in 1973 [43] consists of a 100 × 35 mm rectangular card displaying the question “How is your mood right now?”. Participants indicate their response by marking a point along a horizontal line, where the left end represents the worst mood and the right end the best. The final score is obtained by measuring the distance in millimeters from the left edge to the participant’s mark, providing a simple, sensitive measure of current mood state.

GSE. Originally developed by Matthias Jerusalem and Ralf Schwarzer in 1979, this instrument initially comprising 20 items, was reduced to 10 items in 1981, and was later adapted to 28 languages [173]. The GSE scale has been employed in numerous studies, and its stability has been examined in various longitudinal investigations. Psychometric properties assessed by Scholtz et al. in their 2002 article are presented in Table 1 [173]. In acromegaly, only one study was identified that applied this instrument [118] alongside various other Patient-Reported Outcome Measures (PROMs) in a self-care intervention program.

HADS. A widely utilized instrument in clinical practice and medical research for assessing anxiety and depression among patients receiving hospital care. This self-report scale consists of 14 items initially developed to indicate the likelihood of the presence of anxiety and depressive states in an outpatient clinic setting. It comprises two domains, each with seven items, one for anxiety and the other for depression. Both domains have a scoring range of 0 to 21, with higher scores indicating more significant anxiety and depressive symptoms [118, 174]. Various authors have assessed its applicability in acromegaly, including for the evaluation of self-care programs [118], the development of other questionnaires [19], evaluating the quality of life in patients during follow-up [120], in those with osteoarticular complications [144], in individuals who achieved remission [65], and those undergoing radiotherapy [138].

FSS. A self-report generic questionnaire designed to assess fatigue, the FSS comprises nine items, scored on a scale of 1 to 7 (1 indicating strong agreement and 7 indicating strong disagreement) [175]. In the context of acromegaly, it has been evaluated in a study assessing surgically treated patients [77], where the effects of fatigue on motivation, exercise, physical functioning, work interference, family, and social life in individuals with acromegaly.

PSC. A checklist comprising 55 physical symptoms derived from the DSM-III classification [176], the PSC offers a comprehensive evaluation of various bodily manifestations. These symptoms include most organ systems, with four gender-specific symptoms. Severity ratings, ranging from 0 to 3, are assigned to each symptom across categories such as general/neurological, autonomic, musculoskeletal/pain, gastrointestinal, genital, and hot/cold sensation symptoms [60]. The total symptom score ranges from 0 to 153, where a higher score reflects a more significant number of (severe) physical symptoms experienced in the past week [107]. It has been used to assess illness perceptions and their association with quality of life in patients with acromegaly and long-term biochemical control [106, 107].

### Non-unidomain and non-multidomain questionnaires - generic

BMQ. Developed by Horne et al. [40] to assess patients’ beliefs about medication and adherence to pharmacological treatment, this instrument consists of two sections (BMQ-General and BMQ-Specific), each with two subscales (see Table 1) [40]. Each item is evaluated using a five-point Likert scale of 1 (completely agree) to 5 (completely disagree) [40]. This instrument was applied by Andela et al. in patients with acromegaly in remission [91].

### Advantages and disadvantages of questionnaires

Table 3 summarizes the advantages and disadvantages of the questionnaires. Below is a brief description.

**Table 3:** Advantages and disadvantages of the questionnaires

Questionnaire	Advantages	Disadvantages
AcroQoL	• Specific • Multidomain • Psychometric properties evaluated in patients with acromegaly • Cross-cultural validation • Short application time • Multiple application methods	• None
PASQ	• Specific • Available in multiple languages • Incorporates the measurement of the impact of the treatment	• Unidomain • COSMIN-proposed items not fully assessed
AcroTSQ	• Multidomain • Specific • Psychometric properties evaluated in patients with acromegaly • Multiple application methods • Incorporates the measurement of the impact of the treatment	• None
ACCQ	• Specific • Evaluates common symptoms in acromegaly • Assesses common symptoms of acromegaly	• Unidomain COSMIN-proposed items not fully assessed
SSS	• Specific • Multiple application methods	• Unidomain Evaluated in few studies on acromegaly COSMIN-proposed items not fully assessed
ILQ	• Specific • Multidomain • Multiple application methods • Incorporates the measurement of the impact of the treatment • Psychometric properties evaluated in patients with acromegaly	• Evaluated in few studies of patients with acromegaly
POMS	• Multidomain • Available in multiple languages • Short application time • Assesses common symptoms of acromegaly	• Evaluated in few studies of patients with acromegaly • COSMIN-proposed items not fully assessed
BMQ	• Available in multiple languages • Incorporates the measurement of the impact of treatment	• Unidomain • Applied only by evaluator • Evaluated in few studies on acromegaly • COSMIN-proposed items not fully assessed
IPQ-R	• Multidomain • Available in multiple languages	• Evaluated in few studies on acromegaly • COSMIN-proposed items not fully assessed
EQ-5D-5 L	• Multidomain • Available in multiple languages • Short application time Multiple application methods • Assesses common symptoms of acromegaly	• None
VASM	• Available in multiple languages • Multiple application methods • Easy application: through a visual analog scale • Assesses common symptoms of acromegaly	• Unidomain • Evaluated in few studies of patients with acromegaly COSMIN-proposed items not fully assessed
GSE	• Short application time	• Unidomain COSMIN-proposed items not fully assessed • Evaluated in few studies of patients with acromegaly
LBNQ-Pituitary	• Multidomain • Assesses common symptoms of acromegaly	• Evaluated in few studies of patients with acromegaly • Applied by evaluator COSMIN-proposed items not fully assessed
IPAQ	• Multidomain • Available in multiple languages • Multiple application methods	• COSMIN-proposed items not fully assessed
UCL	• Multidomain • Multiple application methods	• Evaluated in few studies of patients with acromegaly • COSMIN-proposed items not fully assessed
SF-36	• Multidomain • Available in multiple languages • Short application time Multiple application methods • Assesses common symptoms of acromegaly	• None
MFI-20	• Multidomain • Available in multiple languages • Short application time • Assesses common symptoms of acromegaly	• COSMIN-proposed items not fully assessed
HADS	• Short application time • Assesses common symptoms of acromegaly	• Unidomain • COSMIN-proposed items not fully assessed
FSS	• Available in multiple languages	• Unidomain • Evaluated in few studies of patients with acromegaly • COSMIN-proposed items not fully assessed
TSQM-9	• Multidomain • Multiple application methods • Incorporates the measurement of the impact of treatment • Assesses common symptoms of acromegaly	• COSMIN-proposed items not fully assessed
NHP	• Multidomain • Easy application: short answers • Multiple application methods • Assesses common symptoms of acromegaly • Short application time	• COSMIN-proposed items not fully assessed
WOMAC	• Multidomain • Multiple application methods • Assesses common symptoms of acromegaly	• COSMIN-proposed items not fully assessed • Evaluated in few studies of patients with acromegaly • Long application time
AIMS	• Multidomain • Multiple application methods • Evaluates common symptoms in acromegaly	• COSMIN-proposed items not fully assessed • Evaluated in few studies of patients with acromegaly • Long application time
RAND-36	• Multidomain • Multiple application methods • Assesses common symptoms of acromegaly • Available in multiple languages • Short application time	• COSMIN-proposed items not fully assessed guidelines • Evaluated in few studies on acromegaly
PIT-QOL	• Multidomain • Short application time • Multiple application methods • Evaluates common symptoms in acromegaly • Psychometric properties evaluated in patients with acromegaly • Incorporates the measurement of the impact of treatment	• COSMIN-proposed items not fully assesses • Evaluated in few studies of patients with acromegaly
WHOQol-BREF	• Multidomain • Multiple application methods • Available in multiple languages	• COSMIN-proposed items not fully assessed • Evaluated in few studies of patients with acromegaly
PGWBS	• Multidomain • Multiple application methods • Psychometric properties evaluated in patients with acromegaly	• COSMIN-proposed items not fully assessed • Evaluated in few studies of patients with acromegaly
SQ	• Multidomain • Multiple application methods • Assesses common symptoms of acromegaly • Easy application: short answers • Available in multiple languages	• COSMIN-proposed items not fully assessed • Evaluated in few studies on acromegaly
PSC	• Multiple application methods • Evaluates common symptoms in acromegaly	• Unidomain COSMIN-proposed items not fully assessed • Evaluated in few studies of patients with acromegaly
15D	• Multidomain • Short application time • Multiple application methods • Available in multiple languages	• COSMIN-proposed items not fully assessed • Evaluated in few studies of patients with acromegaly

### Advantages

Our appraisal of advantages/disadvantages was guided by established principles for HRQoL/PROM assessment, including (i) HRQoL as a multidimensional construct, (ii) the importance of validity, reliability, and responsiveness, ideally demonstrated in the target population, and (iii) feasibility considerations such as respondent burden and applicability across languages and cultures. [23, 24, 30, 31]

All three authors converged on the importance of differentiating between generic and disease-specific instruments when evaluating HRQoL in acromegaly. Disease-specific instruments were considered advantageous because they are designed to capture impacts that are particularly relevant to acromegaly (symptoms, appearance-related concerns, and treatment burden), which supports content relevance for this population. [4, 7, 25]

The number of domains assessed was another criterion considered. Since HRQoL is inherently multidimensional, instruments capturing more than one core domain were considered advantageous for comprehensive HRQoL assessment. [23, 33] Among those meeting this criterion, some assess all proposed domains (e.g., AcroQoL, SF-36, NHP, AIMS, RAND-36, PIT QOL, WHOQOL-BREF, PGWBS, 15D, ILQ). Others evaluate only selected domains (e.g., AcroTSQ, POMS, IPQ-R, EQ-5D-5 L, LBNQ-Pituitary, IPAQ, UCL, MFI-20, TSQM-9, WOMAC, SQ); however, we did not treat “covering all four domains” as an additional advantage because domain prioritization may vary depending on study aims and clinical context, and because unidomain/partial-domain instruments are often used as complementary measures rather than stand-alone HRQoL tools. [24, 33]

As previously noted and depicted in Table 1, several scales have been subject to psychometric property assessments; however, these were considered advantageous only when explicitly evaluated in individuals with acromegaly, consistent with recommended approaches for PROM evaluation in the target population. [30, 31] Cross-cultural validation and availability in multiple languages were also considered strengths because they support broader applicability and comparability across settings. [15, 24]

The authors also found flexibility in administration (self-reported and interviewer-administered options) advantageous. Most authors (JECS, OFAR) recommended a completion time of 10 minutes or less, and all authors agreed that ease of administration is an advantage, particularly when response formats are simple (e.g., yes/no) or based on visual analogue scales. [24] Furthermore, instruments incorporating treatment-impact measurement on HRQoL were considered advantageous given the chronic nature of acromegaly and the relevance of treatment burden and satisfaction to patient-centered outcomes. [4, 7, 35, 38].

### Disadvantages

All authors unanimously identified the assessment of a single domain as a major limitation of certain questionnaires, as unidimensional instruments do not comprehensively capture the multidimensional nature of HRQoL and are therefore less suitable as stand-alone outcome measures.

Another important limitation was the absence of studies evaluating all recommended psychometric properties according to COSMIN guidelines, which may hinder robust interpretation of results and limit confidence in the quality of some PROMs. Additional disadvantages included prolonged administration time (exceeding 10 minutes), instruments that require interviewer administration only, and limited use or evaluation in studies involving patients with acromegaly. Further disadvantages are detailed in Table 3. All authors unanimously identified the assessment of a single domain as a significant drawback for instruments used as stand-alone HRQoL measures, as they do not comprehensively address HRQoL’s multidimensional construct; such tools may be better positioned as adjunct symptom-focused measures. [23, 33]

Another limitation is the absence of studies assessing key psychometric properties according to current recommendations, which may hinder the ability to draw definitive conclusions about the quality of some PROMs. [30, 31] Moreover, prolonged administration time (exceeding 10 minutes), instruments applied only by an evaluator (not self-administered), and limited evaluation specifically in acromegaly populations were considered additional limitations. Further disadvantages are detailed in Table 3. [24, 30, 31]

## Discussion

This scoping review details all the PROMs used in patients with acromegaly to assess their HRQoL. We provide a synthesis of their psychometric properties, categorize them based on domains, and outline their advantages and disadvantages as considered by the authors.

We conclude by consensus that to assess HRQoL in acromegaly, instruments should be disease-specific, multidomain, brief, easy to administer, available in multiple languages, psychometrically validated in patients with acromegaly, and cross-culturally validated to assess treatment impact. Among the reviewed instruments, AcroQoL emerges as the most suitable meeting these criteria, and we recommend its use in future studies.

The domain-based classification proposed in this review represents a novel contribution that may help overcome the current heterogeneity in the use and interpretation of PROMs in acromegaly. By organizing instruments according to specificity (generic vs. disease-specific) and dimensionality (uni- vs. multidomain), this framework facilitates the comparison of psychometric robustness and clinical applicability across tools. Unlike prior classifications that grouped instruments only by scope or target population, our approach highlights both conceptual coverage and measurement depth-two key aspects to ensure that PROMs truly reflect the multidimensional nature of HRQoL in acromegaly. This structure may guide clinicians and researchers in selecting tools that balance feasibility with comprehensiveness, ultimately supporting greater standardization and comparability across studies. This conceptual structure not only reflects the multidimensional construct of HRQoL endorsed by COSMIN and prior theoretical models but also provides a practical taxonomy that can be replicated in other rare endocrine disorders.

Other authors have mentioned several other instruments used in patients with acromegaly, albeit with different objectives. Following a systematic search, Camerini et al. (215) described specific acromegaly questionnaires used in various studies. Notably, AcroQoL was highlighted for its user-friendly nature, conciseness, and reliance on self-report, aligning with the findings in the present review. Other identified questionnaires include ACRODAT, SAGIT, and ACROSCORE, each playing a pivotal role in acromegaly management by facilitating early symptom identification and monitoring disease activity during follow-up. In contrast to these studies, ours did not include instruments such as ACRODAT, SAGIT, and ACROSCORE. These focus on objective outcomes (e.g., biochemical control) that, while crucial in acromegaly, are classified as essential for clinicians (CRO, Clinician Reported Outcomes) rather than for patients (PRO), diverging from the central objective of this article [135].

In a systematic review and meta-analysis by Van der Meulen et al. in patients with acromegaly or growth hormone deficiency [177]), several PROMs were evaluated to identify their properties (validity, quality of reporting, similarities, and discrepancies of PROs with biochemical outcomes). The review revealed that numerous PROMs used in studies involving patients with acromegaly lack validation, posing challenges in result comparison across studies. Similarly, the quality of reporting for most of these PROMs did not align with current guidelines. Consistent with these findings, we observed that many of the questionnaires lacked studies evaluating the criteria proposed by the COSMIN methodology, particularly regarding measurement error and responsiveness, which are essential for interpretability and detecting changes over time. This shortcoming could hinder reviewers from drawing clear conclusions and making evidence-based recommendations on the quality of PROMs, thereby limiting their selection for research and clinical practice [31]. A distinction from the work mentioned above is that the present study identified other acromegaly-specific questionnaires that offer particular advantages. These include the SSS, which assesses symptom severity; AcroTSQ and ILQ, which capture the impact of treatment on HRQoL; and ACCQ, which explores the implications of manifestations and comorbidities caused by acromegaly on HRQoL. The significance of this lies in the increasing recognition, by different study groups in acromegaly, of the importance of these specific PROMs, leading to efforts in validating and implementing them in research studies and clinical practice.

From a clinical perspective, our results emphasize the need to balance the use of disease-specific and generic tools. Disease-specific instruments, such as AcroQoL or AcroTSQ, are better suited for capturing subtle changes related to treatment and symptom burden, thus informing individualized, patient-centered care. In contrast, generic instruments like the SF-36 or EQ-5D facilitate comparisons across diseases and support health-economic evaluations. Integrating both approaches, using a generic PROM for benchmarking and a disease-specific one for clinical follow-up, may provide a more comprehensive understanding of patient well-being.

However, several barriers may limit the broader adoption of PROMs in acromegaly. These include the lack of standardization in instrument selection, limited availability of culturally adapted versions, and practical constraints such as time burden during clinical visits. To promote implementation, shorter instruments with electronic formats or integrated platforms could be prioritized, provided their psychometric robustness is ensured. Training clinicians in interpreting PROM results and embedding them into shared decision-making processes may further enhance their utility in routine care.

Other studies, such as the one by Broersen et al. [135], have also mentioned some PROMs used in individuals with acromegaly. Unlike the present study, the objective of these authors was to evaluate the effect of acromegaly treatment on patients’ symptoms and HRQoL. However, they did not describe all the PROMs used in the studies, nor did they discuss their utility for other relevant outcomes in patients with acromegaly. This approach was considered in the present study.

The above highlights the importance of evaluating PROMs. Using them in both clinical and research settings is crucial, as they gauge patients’ perspectives on a given condition or chronic disease. Furthermore, they hold relevance for physicians and are instrumental in public health initiatives. Their implementation is fundamental in the context of acromegaly, as they can capture subjective outcomes that other traditionally used tools may lack the capacity to assess [4, 178]. Additionally, these subjective outcomes, encompassing symptom improvement and HRQoL, are considered pivotal goals for both patients and clinicians when assessing treatment success. This highlights the need for instruments that adequately evaluate these outcomes [135]. Finally, some PROMs have become a means to assess the effectiveness of Self-Management Interventions (SMIs) in patients with acromegaly, showing promising results in improving psychological aspects in these patients [118].

To our knowledge, this is the first review that comprehensively describes all PROM instruments evaluated in individuals with acromegaly. It also assesses the adherence of studies on patients with acromegaly to guidelines for reporting PROM results. Finally, it proposes a classification of PROMs based on four domains considered relevant for assessing HRQoL, which has not been the focus of similar studies. We consider that this novel classification proposal based on domains and specificity will help standardize the questionnaires, reducing heterogeneity and facilitating cross-study comparisons. Ultimately, this may improve PROM selection for both research and clinical applications, enhancing the integration of patient-reported outcomes into evidence-based care for acromegaly.

The current review has some limitations. The lack of global standardization in defining the aspects that PROMs must meet to assess HRQoL, especially in acromegaly, may have affected the search process, implying that some instruments used by other authors may not have been identified in the review. We therefore recommend adopting clear evaluation criteria for PROMs, such as those proposed in this study, for HRQoL assessment in acromegaly. Another of the objectives of this review was to synthesize information on questionnaire validation. Unfortunately, the search highlighted a lack of uniformity and adherence to guidelines in reporting results, limiting the achievement of this goal. Finally, establishing the advantages and disadvantages of each instrument was complex given the heterogeneity in approaches and contexts in which they have been applied.

## Conclusions

This review addresses the use of PROMs to assess acromegaly patients’ HRQoL, emphasizing the importance of using instruments that are disease-specific, multidomain, cross-cultural validated, available in multiple languages, psychometrically validated in individuals with acromegaly, easy to apply, brief, with various application methods and finally, that assess common symptoms of acromegaly and incorporate the impact of treatment. A novel domain-based classification is proposed to be included in future research to help overcome certain limitations of these instruments, including the lack of standardization and quality in result presentation.

This review is a valuable resource for future research and clinical practice in acromegaly HRQoL assesment.

## Electronic supplementary material

Below is the link to the electronic supplementary material.

Supplementary Material 1

## Data Availability

No data was collected in the development of this manuscript.
