# Supplementary material for: Quality of life questionnaires in patients with acromegaly: a scoping review
Source: J Patient Rep Outcomes. 2026 Mar 11;10:119. doi: 10.1186/s41687-026-01022-3 (PMC13369093; doi:10.1186/s41687-026-01022-3)
Supplement: Supplementary file 1 — Supplementary Material 1 [file 41687_2026_1022_MOESM1_ESM.docx]

# **Supplementary material**

## Table S1. Search strategy

| **Source** | **Strategy** | **Results** | **Date** |
| --- | --- | --- | --- |
| MEDLINE (Pubmed) | ("acromegaly"[Title/Abstract]) AND ((quality of life[Title/Abstract]) OR (QoL[Title/Abstract]) OR (patient satisfaction[Title/Abstract]) OR (patient reported outcome[Title/Abstract])) AND ((questionnaire[Title/Abstract]) OR (questionnaires[Title/Abstract])) | 130 records | September/2022 |
| EMBASE | ('acromegaly'/exp OR 'acromegaly') AND (('quality of life'/exp OR 'quality of life' OR qol OR patient) AND satisfaction OR patient) AND reported AND outcome AND (questionnaire OR questionnaires) | 32 records | September/2022 |
| CINAHL | MM Acromegaly OR TI Acromegaly OR TX Acromegaly  AND MM "quality of life" OR TI "quality of life" OR TX "quality of life" AND MM Questionnaires OR TI Questionnaires OR TX Questionnaires | 112 records | November/22 |

## Table S2. Underused patient-reported outcome measures (PROMs) in acromegaly and main reasons for limited validation

| **Instrument** | **Type** | **Structure** | **Frequency (approx.)** | **Psychometric validation in acromegaly** | **Main reason for limited validation** | **Example of use / context** | **Comment** |
| --- | --- | --- | --- | --- | --- | --- | --- |
| **FSS** | Generic | Unidomain | 1 | No | Focused only on fatigue; not validated in acromegaly | Used post-surgery to assess fatigue impact (77) | Complementary PROM |
| **HADS** | Generic | Unidomain | 5 | Partial | Evaluates anxiety/depression only; lacks acromegaly validation | Used in self-care programs and follow-up studies (118,140) | Broadly validated in other diseases |
| **VAS-Mood** | Generic | Unidomain | 1 | No | One-dimensional; not validated for acromegaly | Used in psychological self-care interventions (118) | Simple but limited scope |
| **IPAQ (IPA)** | Generic | Multidomain | 1 | No | Developed for chronic conditions; no adaptation to acromegaly | Used in self-management programs (118) | Measures autonomy/participation rather than QoL |
| **UCL** | Generic | Multidomain | 1 | No | Focuses on coping strategies; lacks HRQoL integration | Applied in self-care interventions (118) | Psychological support measure |
| **BMQ** | Generic | Unidomain | 1 | No | Focuses on medication beliefs, not HRQoL | Used in remission patients (91) | Captures adherence, not QoL |
| **PSC** | Generic | Unidomain | 2 | No | Symptom checklist, not designed as QoL PROM | Used to assess illness perceptions (106,107) | Useful as secondary outcome |
| **FSS** | Generic | Unidomain | 1 | No | Focused solely on fatigue; lacks acromegaly-specific validation | Used post-surgery (77) | Narrow focus |
| **WOMAC** | Generic | Multidomain | 1 | No | Specific for osteoarthritis; not adapted to acromegaly | Used for musculoskeletal complications (140) | Context-specific, limited scope |
| **AIMS** | Generic | Multidomain | 1 | No | Designed for arthritis; no acromegaly validation | Used for musculoskeletal assessment (75) | Overlaps with WOMAC |
| **PGWBS** | Generic | Multidomain | 3 | Partial | Psychometric testing outside acromegaly; few studies | Used in radiotherapy and remission cohorts (37,72) | Promising but underused |
| **SQ** | Generic | Multidomain | 1 | No | Not validated for acromegaly; limited to psychological distress | Used in lanreotide-treated patients (159) | Historical but narrow |
| **15D** | Generic | Multidomain | 1 | Partial | Generic elderly-population scale; few acromegaly applications | Used in surgically treated patients (162) | Could complement generic HRQoL measures |

Table S2 summarizes the patient-reported outcome measures (PROMs) identified as underused in acromegaly. Instruments are classified by type and structure, with frequency of use derived from the number of studies identified in the scoping review (see Table 1). “Psychometric validation in acromegaly” indicates whether measurement properties have been evaluated specifically in this population. The main reasons for limited validation include unidimensional scope (e.g., symptom-specific focus), administrative burden, lack of disease-specific or cross-cultural validation, and preferential use of widely validated generic tools.
